# Multiple bacteria associated with the more dysbiotic genitourinary microbiomes in patients with type 2 diabetes mellitus

**DOI:** 10.1038/s41598-021-81507-x

**Published:** 2021-01-19

**Authors:** Hua Zha, Fengping Liu, Zongxin Ling, Kevin Chang, Jiezuan Yang, Lanjuan Li

**Affiliations:** 1 State Key Laboratory for Diagnosis and Treatment of Infectious Diseases, Collaborative Innovation Center for Diagnosis and Treatment of Infectious Diseases, National Clinical Research Center for Infectious Diseases, The First Affiliated Hospital, Zhejiang University School of Medicine, 79 Qingchun Road, Hangzhou, 310000 China; 2 School of Biological Sciences, The University of Auckland, Auckland, New Zealand; 3 Institute of Marine Science, The University of Auckland, Auckland, New Zealand; 4 School of Medicine, Jiangnan University, Wuxi, China; 5 Department of Statistics, The University of Auckland, Auckland, New Zealand

**Keywords:** Microbiology, Bacteria, Microbial communities, Diseases, Urogenital diseases

## Abstract

Type 2 diabetes mellitus (T2DM) influences the human health and can cause significant illnesses. The genitourinary microbiome profiles in the T2DM patients remain poorly understood. In the current study, a series of bioinformatic and statistical analyses were carried out to determine the multiple bacteria associated with the more dysbiotic genitourinary microbiomes (i.e., those with lower dysbiosis ratio) in T2DM patients, which were sequenced by Illumina-based 16S rRNA gene amplicon sequencing. All the genitourinary microbiomes from 70 patients with T2DM were clustered into three clusters of microbiome profiles, i.e., Cluster_1_T2DM, Cluster_2_T2DM and Cluster_3_T2DM, with Cluster_3_T2DM at the most dysbiotic genitourinary microbial status. The three clustered T2DM microbiomes were determined with different levels of alpha diversity indices, and driven by distinct urinalysis variables. OTU12_*Clostridiales* and OTU28_*Oscillospira* were likely to drive the T2DM microbiomes to more dysbiotic status, while OTU34_*Finegoldia* could play a vital role in maintaining the least dysbiotic T2DM microbiome (i.e., Cluster_1_T2DM). The functional metabolites K08300_ribonuclease E, K01223_6-phospho-beta-glucosidase and K00029_malate dehydrogenase (oxaloacetate-decarboxylating) (NADP+) were most associated with Cluster_1_T2DM, Cluster_2_T2DM and Cluster_3_T2DM, respectively. The characteristics and multiple bacteria associated with the more dysbiotic genitourinary microbiomes in T2DM patients may help with the better diagnosis and management of genitourinary dysbiosis in T2DM patients.

## Introduction

Type 2 diabetes mellitus (T2DM) is a global health problem^1^. It contributes to the increasing rate of non-communicable diseases in both developed and developing counties^2^, and could lead to mortalities^3^. The associations between gut microbiome and T2DM have been well studied^4–9^, however, genitourinary microbiomes in T2DM patients were relatively understudied.

Recently, the genitourinary microbiomes of T2DM cohorts have been studied to achieve different objectives^10–12^. *Actinobacteria*, *Collinsella*, *Desulfovibrio*, *Enterobacteriaceae*, *Flavobacteria*, *Flavobacteriales*, *Lactobacillus* and *Porphyromonas* were capable of distinguishing the genitourinary microbiomes of T2DM patients from those of healthy subjects^9,10^. *Lactobacillus* and *Prevotella* were predominant in all the four cohorts of T2DM patients in a previous study^12^: (1) T2DM only, (2) T2DM and hypertension, (3) T2DM and hyperlipidemia, and (4) T2DM, hypertension and hyperlipidemia. *Bifidobacteriaceae*, *Thermaceae* and *Shuttleworthia* could contribute to the presence of interleukin-8 in the urine of T2DM patients^11^.

The microbiome profiles of the diseased cohorts in different disease studies have attracted increasing scientific attention^13–16^. Cirrhosis dysbiosis ratio was associated with the severity of liver cirrhosis^17^, and the dysbiosis ratio of microbiome has been used to evaluate the dysbiotic statuses of different microbiome profiles^18,19^, i.e., a greater dysbiosis ratio represented a less dysbiotic status. We hypothesised that there was a specific genitourinary microbiome profile of the recruited T2DM cohort was at a more dysbiotic status. In the current study, we aim to (1) determine the characteristics and dysbiotic statuses of different genitourinary microbiome profiles in the T2DM patients; (2) investigate the phylotypes associated with the more dysbiotic genitourinary microbiome in T2DM patients.

## Methods and materials

### Recruitment of subjects and sample collection

Seventy female patients with T2DM and 70 healthy female subjects were recruited in the present study, and the selection criteria of T2DM patients were described in our previous study^12^. Briefly, both patients and healthy individuals aged between 26 and 85 years old (Supplementary Fig. S1), and had similar body mass indices. Individuals with the following attributes were not included in this study: intake of antibiotics, probiotics, prebiotics or synbiotics in the past three months; menstruation during the study; with relevant genitourinary diseases or abnormal conditions.

The urine samples were collected by using a modified midstream urine collection technique involving disinfection and a four-tube collection method^12^. Briefly, four opened 50-ml sterile centrifuge tubes were prepared with lids upwards. The participants were pants off and squatted on a squat toilet. Antiseptic cotton balls were handled by disinfected hand to clean the far labial fold and then the near labial fold. The labia were held apart and the participant urinated into the four tubes (tubes 1–4) in order. The urine in tube 2 and 3 were aliquoted for three subsequent analyses: 15 ml for urinalysis, 1 ml for urine culture and 40 ml for Illumina sequencing.

Written informed consent was taken from all the participants prior to enrolment, and the study protocol was approved by the Institutional Review Board of the First Affiliated Hospital, School of Medicine, Zhejiang University (Zhejiang, China). The study was carried out under relevant guidelines and regulations (Declaration of Helsinki).

### Molecular methods

Bacterial genomic DNA of all the urine samples from all the T2DM patients and healthy subjects, as well as a DNA-free water sample (blank control), was extracted by Liu, et al.^12^, followed by the amplifications of barcoded 16S rDNA primers targeting the V3–V4 regions of all the extracted DNA samples. The amplicons were purified, quantified, pooled, and then sequenced on the Illumina MiSeq instrument using 2-by-300 bp chemistry^12^.

### Processing of the sequencing data

The raw sequencing data were processed as described by Liu et al.^12^, including sequencing merge, chimera check, quality filtering and taxonomy assignment by using QIIME (version 1.9.0). The sample rarefaction was performed using the phyloseq package in R (version 3.6.1).

### Microbial dysbiotic status of genitourinary microbiomes in T2DM and healthy cohorts

Linear discriminant analysis (LDA) effect size (LEfSe) was carried out in a program run by the Huttenhower lab to determine the OTUs associated with the genitourinary microbiomes of T2DM patients or healthy subjects.

The microbial dysbiosis ratio (MDR), i.e., the abundance ratio of “good and bad taxa”, was used to help determine the dysbiotic status of microbiomes in different disease studies^17,20^. In the present study, genitourinary MDR was defined as the abundance ratio of genitourinary OTUs associated with healthy cohort (n = 70) and genitourinary OTUs associated with T2DM cohort (n = 70). The genitourinary MDRs of the genitourinary microbiomes in healthy and T2DM cohorts were transformed in log10 to satisfy the assumptions of normal distribution and equal variance, before being compared by a *t* test.

A spearman’s test was performed to determine the correlations between the multiple variables and diversity indices (i.e., observed species, Shannon and Pielou indices) in all T2DM patients.

### Clustering of the genitourinary microbiomes in T2DM patients

Partition around medoids (PAM) clustering analysis has been used in different disease studies for achieving different objectives^15,21,22^. In the current study, PAM analysis was carried out to cluster the genitourinary microbiomes from all the 70 T2DM patients, after the optimal number of clusters was determined by the average silhouette method^23^. Three clusters of T2DM microbiomes were determined, i.e., Cluster_1_T2DM, Cluster_2_T2DM and Cluster_3_T2DM.

### Correlations between urinalysis variables and T2DM microbiome in each of the three clusters

The urinalysis variables with the significant effects on the genitourinary microbiomes were determined with one-way ANOVA^24^, and the variables with *P* < 0.05 were selected for the distance-based redundancy analysis (db-RDA) in Primer v7 (Primer-e Ltd., New Zealand).

### Comparisons of T2DM associated OTUs between the three clustered T2DM microbiomes

To determine whether the T2DM associated OTUs had different associations with the three clusters of T2DM microbiomes, the average abundances of T2DM associated OTUs in the three clustered T2DM microbiomes were compared with Kruskal–Wallis tests. Mann–Whitney tests were carried out for the pairwise comparisons, and Bonferroni correction was used for adjusting the *P* values. The average abundances of the T2DM associated OTUs (that were more abundant in one or two clustered microbiomes) in the three clustered T2DM microbiomes were visualized in a heatmap in R version 3.6.1.

### Differences between the three clustered T2DM microbiomes

Permutation analysis of variance (PERMANOVA) was applied in R by using adonis command^25^, to compare the three clustered genitourinary microbiomes from the T2DM patients. Similarity percentage (SIMPER) analysis was performed in Primer v7 to determine the similarities within each of the three clustered T2DM microbiomes, as well as the dissimilarities between the three clustered microbiomes.

The observed species (richness) of the three clustered T2DM microbiomes were transformed in square root to satisfy the assumptions of normal distribution and equal variance. A one-way analysis of variance (ANOVA) was performed to compare the transformed observed species, Shannon index (both richness and evenness) and Pielou index (evenness) of the three clustered T2DM microbiomes. *T* tests were used for the pairwise comparisons, with Bonferroni correction for adjusting the *P* values.

Genitourinary MDRs of the three clustered T2DM microbiomes were transformed in log10 before being compared with one-way ANOVA. *T* tests were carried out for the pairwise comparisons of genitourinary MDRs, with Bonferroni correction for adjusting the *P* values.

A LEfSe analysis was used to determine the representative OTUs associated with each of the three clustered T2DM microbiomes, with LDA threshold over 2.5^26,27^. Pairwise SIMPER analyses were conducted to determine the OTUs contributing most to the dissimilarities between the most dysbiotic microbiomes (i.e., Cluster_3_T2DM) and each of the less dysbiotic T2DM microbiomes (i.e., Cluster_1_T2DM and Cluster_2_T2DM), with a cut-off of 70%^28,29^. The Venny program version 2.1^30^ was used to determine whether any representative OTUs associated with Cluster_3_T2DM could also contribute most to the dissimilarities between Cluster_3_T2DM and Cluster_1_T2DM/Cluster_2_T2DM.

Kruskal–Wallis test was used to compare the multiple variables of the T2DM patients in the three clusters. Mann–Whitney test was performed for pairwise comparisons of marriage times in the three clustered patients.

### Network and fragmentation analyses

Co-occurrence Network inference (CoNet) program was used to determine the correlations of the OTUs within each of the three clustered T2DM microbiomes, based on an ensemble of correlation measures as described by Faust, et al.^31^. The detailed manipulations followed Wagner Mackenzie, et al.^21^. Five coefficients, i.e., Mutual Information, Pearson, Bray Curtis, Spearman and Kullback–Leibler dissimilarities for the ensemble inference and the greatest 1000 positive and negative correlations were determined. The permutations were used to compute the preliminary individual method-specific *P* values, before computing the bootstraps by merging all the initial *P* values into a final *P* value by using Brown's method^32^.

Network fragmentation calculations and generation of a null distribution were carried out in R using the package igraph^33^, to determine the gatekeeper(s) in each of the three clustered T2DM microbiomes that could cause collapse of the corresponding clustered microbiomes. The details of this approach were described in Wagner Mackenzie, et al.^21^. A null distribution of fragmentation scores was created from 10,000 randomly constructed networks with identical node and edge distributions to the original network. Statistical significance was defined as the number of times a fragmentation score greater than that resulting from the removal of the OTU within the null distribution.

### Correlations between urinalysis variables and representative OTUs in each of the three clustered T2DM microbiomes

The correlations between urinalysis variables and representative OTUs in each of the three clustered T2DM microbiomes were determined by CoNet analysis and visualized in Cytoscape software version 3.7.2^34^. The detailed procedures of CoNet analysis were described above following Wagner Mackenzie et al.^21^.

### Functional metabolites associated with each of the three clustered T2DM microbiomes

The functional metabolites for the three clustered T2DM microbiomes were determined with a Tax4fun package in R^35^. A LEfSe analysis was performed to determine the functional metabolites associated with each of the three clustered T2DM microbiomes. Those functional metabolites with LDA score over 2.5 and consistently significant across either of the three clustered T2DM microbiomes were determined being associated with the corresponding clusters of T2DM microbiomes.

## Results

### Microbial dysbiotic status of genitourinary microbiomes in T2DM and healthy cohorts

LEfSe analysis determined that 27 OTUs were associated with T2DM, and 71 OTUs associated with healthy cohort (Supplementary Table S1). The genitourinary MDR was greater in the genitourinary microbiomes of healthy subjects (median 34 ± SE 129) compared with T2DM patients (median 0.8 ± SE 0.9) (*t* test, *P* < 0.001), suggesting the genitourinary microbiomes in T2DM patients were at more dysbiotic status compared with those of healthy cohort at baseline.

No variable was determined as confounder to the microbiome diversity indices in T2DM cohort (− 0.37 < all correlation coefficient < 0.32) (Supplementary Table S2).

### Clustering of genitourinary microbiomes in T2DM patients

**Figure 1:**
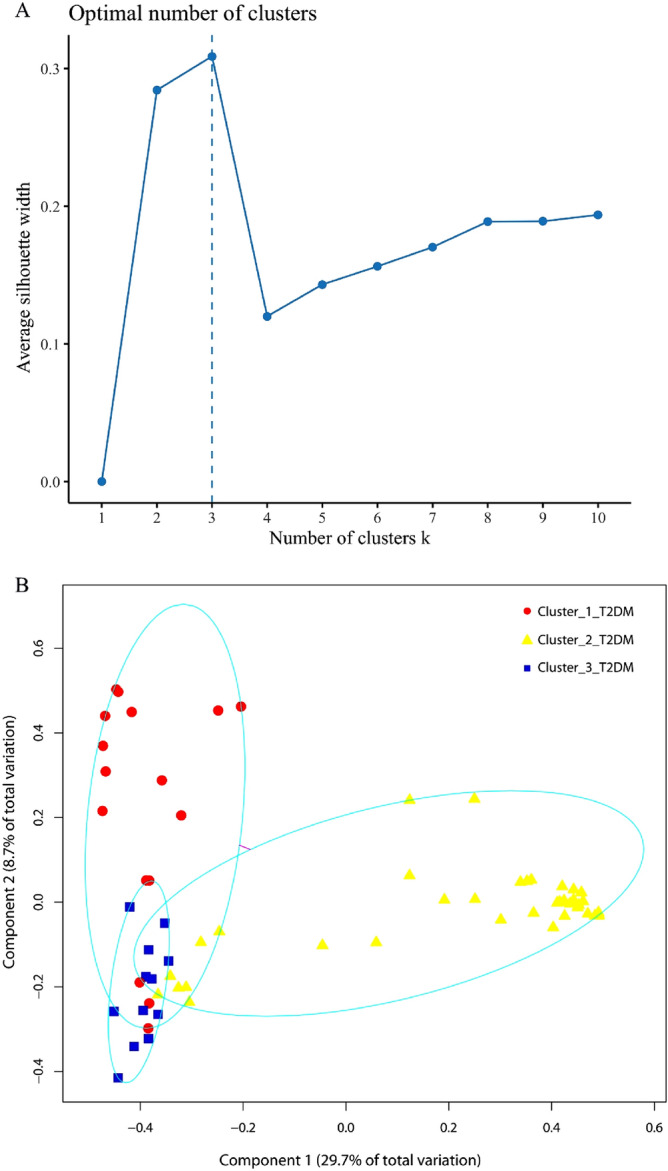
Clustering of the genitourinary microbiomes in T2DM patients by (**A**) average silhouette analysis and (**B**) partition around medoids clustering analysis, into three clusters of T2DM microbiomes, i.e., Cluster_1_T2DM, Cluster_2_T2DM and Cluster_3_T2DM. Note: the analyses were performed in R version 3.6.1.

Silhouette analysis determined three as the most optimal number for clustering the T2DM microbiomes (Fig. 1A). The three clustered T2DM microbiomes, i.e., Cluster_1_T2DM, Cluster_2_T2DM and Cluster_3_T2DM, contained 16, 42 and 12 individual T2DM microbiomes, respectively (Fig. 1B). The three clusters of T2DM microbiomes and healthy microbiomes were determined with different abundant families (Supplementary Fig. S2).

### Correlations between urinalysis variables and T2DM microbiomes in each of the three clusters

**Figure 2:**
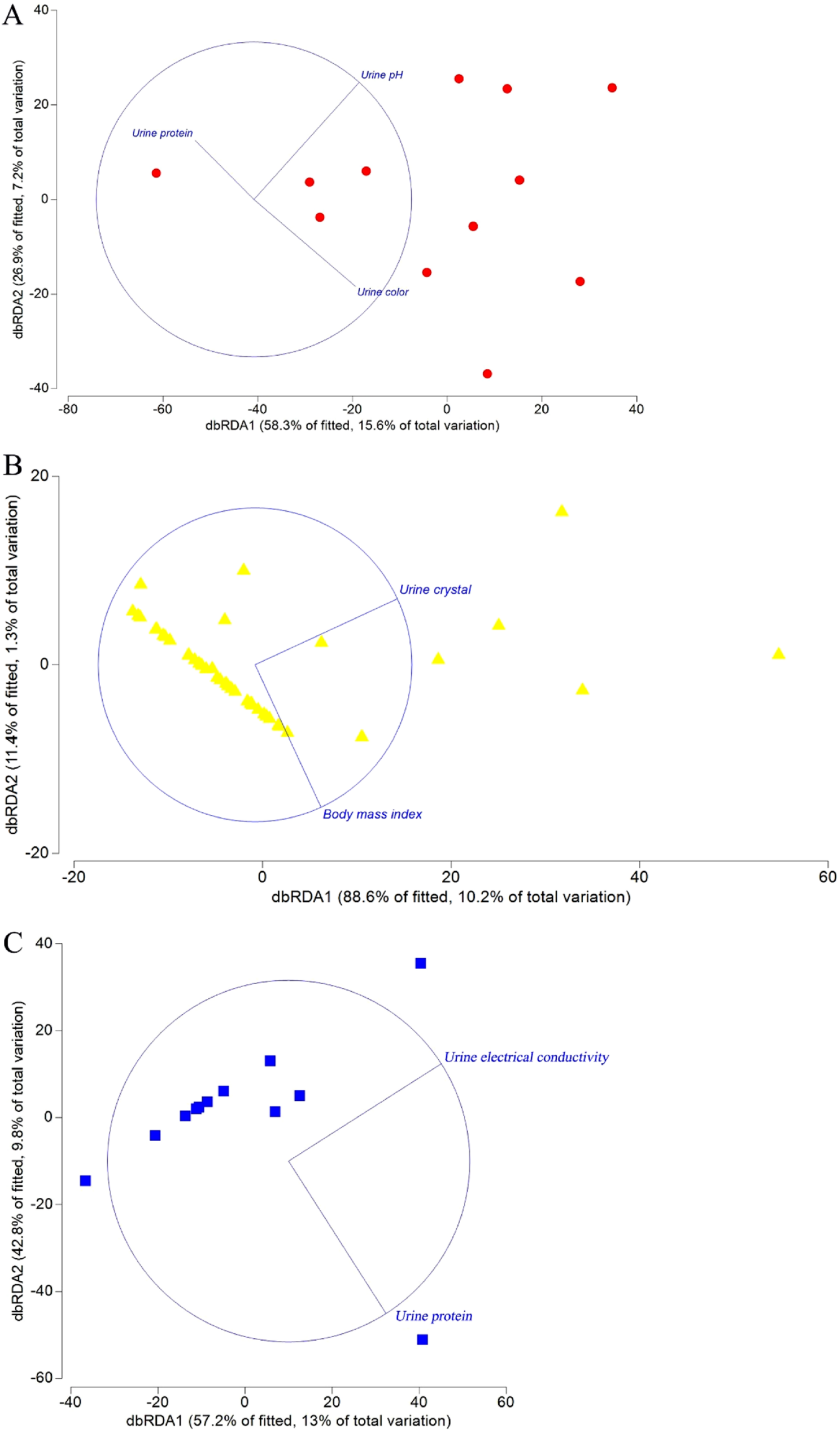
The urinalysis variables determined to affect the three clustered T2DM microbiomes, i.e., (**A**) Cluster_1_T2DM (red), (**B**) Cluster_2_T2DM (yellow) and (**C**) Cluster_3_T2DM (blue). Note: the analysis was carried out in a PRIMER7 software.

Different urinalysis variables were determined to influence the different microbiome profiles of T2DM patients according to the db-RDA results. Urine color, urine pH and urine protein greatly influenced the Cluster_1_T2DM (Fig. 2A). Likewise, BMI and urine crystal had great influences on Cluster_2_T2DM (Fig. 2B), while electrical conductivity and urine protein greatly influenced Cluster_3_T2DM (Fig. 2C).

### Comparisons of T2DM associated OTUs between the three clustered T2DM microbiomes

**Figure 3:**
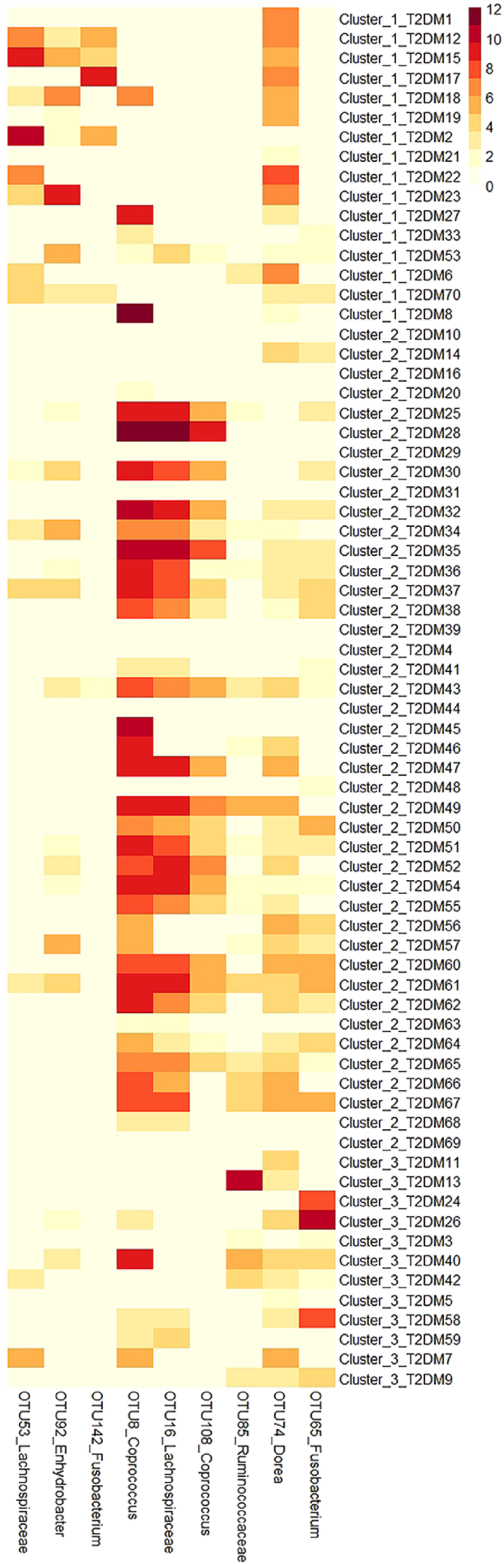
Distribution of T2DM associated OTUs in the three clustered T2DM patients visualized in a heatmap. Note: the abundance of the OTUs were transformed in log_2_(raw read + 1) in the heatmap, and the heatmap figure was done in R version 3.6.1.

The average abundances of T2DM associated OTUs in the three clustered T2DM microbiomes were compared to determine their associations with the different clusters of T2DM microbiomes. A total of 13 OTUs associated with T2DM were determined with different average abundances between the three clustered T2DM microbiomes (Kruskal–Wallis test, all *P* < 0.05), nine of which were more abundant in one or two cluster(s) of T2DM microbiomes (Mann–Whitney test, all *P* < 0.05). OTU53_*Lachnospiraceae*, OTU92_*Enhydrobacter* and OTU142_*Fusobacterium* were more abundant in Cluster_1_T2DM (Fig. 3). OTU8_*Coprococcus*, OTU16_*Lachnospiraceae* and OTU108_*Coprococcus* were more abundant in Cluster_2_T2DM, while OTU85_*Ruminococcaceae* was more abundant in Cluster_3_T2DM (Fig. 3). OTU74_*Dorea* had greater abundances in both Cluster_1_T2DM and Cluster_3_T2DM, while OTU65_*Fusobacterium* had greater abundances in both Cluster_2_T2DM and Cluster_3_T2DM (Fig. 3).

### Differences between the three clustered T2DM microbiomes

PERMANOVA showed a significant difference between the three clustered T2DM microbiomes (R^2^ = 0.27, *P* < 0.001). The similarity within Cluster_2_T2DM (SIMPER average similarity = 46%) was higher than those within Cluster_1_T2DM (SIMPER average similarity = 16%) or Cluster_3_T2DM (SIMPER average similarity = 10%). The dissimilarities between the three clustered T2DM microbiomes were all over 90%.

There were significant differences in the observed species, Shannon and Pielou indices between the three clustered T2DM microbiomes (one-way ANOVA, all *P* < 0.001). The observed species and Shannon index were both largest in Cluster_1_T2DM compared with Cluster_2_T2DM and Cluster_3_T2DM (*t* test, all *P* < 0.02) (Table 1). The Pielou index of Cluster_1_T2DM was significantly larger than that of Cluster_2_T2DM (*t* test, *P* < 0.001), and slightly larger than Cluster_3_T2DM (*t* test, *P* = 0.053) (Table 1).

**Table 1:** Comparisons of observed species (richness), Shannon index (both richness and evenness) and Pielou index (evenness) in the three clusters of T2DM microbiomes, i.e., Cluster_1_T2DM, Cluster_2_T2DM and Cluster_3_T2DM.

Alpha diversity	Cluster_1_T2DM	Cluster_2_T2DM	Cluster_3_T2DM
Observed species	444 ± 70^a^	150 ± 13^b^	237 ± 39^b^
Shannon index	3.9 ± 0.36^a^	1.75 ± 0.14^b^	2.7 ± 0.3^c^
Pielou index	0.64 ± 0.05^a^	0.35 ± 0.02^b^	0.5 ± 0.04^a^

Results were represented in mean ± S.E., and the groups with different alphabets represented significant difference between the clustered T2DM microbiomes.

The genitourinary MDR was lower in Cluster_3_T2DM (Median 0.05 ± SE 2.38) compared with Cluster_1_T2DM (5.33 ± 2.63) and Cluster_2_T2DM (0.92 ± 4.15) (*t* test, both *P* < 0.01), while it was similar between Cluster_1_T2DM and Cluster_2_T2DM (*t* test, *P* = 0.425). This suggests that Cluster_3_T2DM was at more dysbiotic status compared to Cluster_1_T2DM and Cluster_2_T2DM.

**Figure 4:**
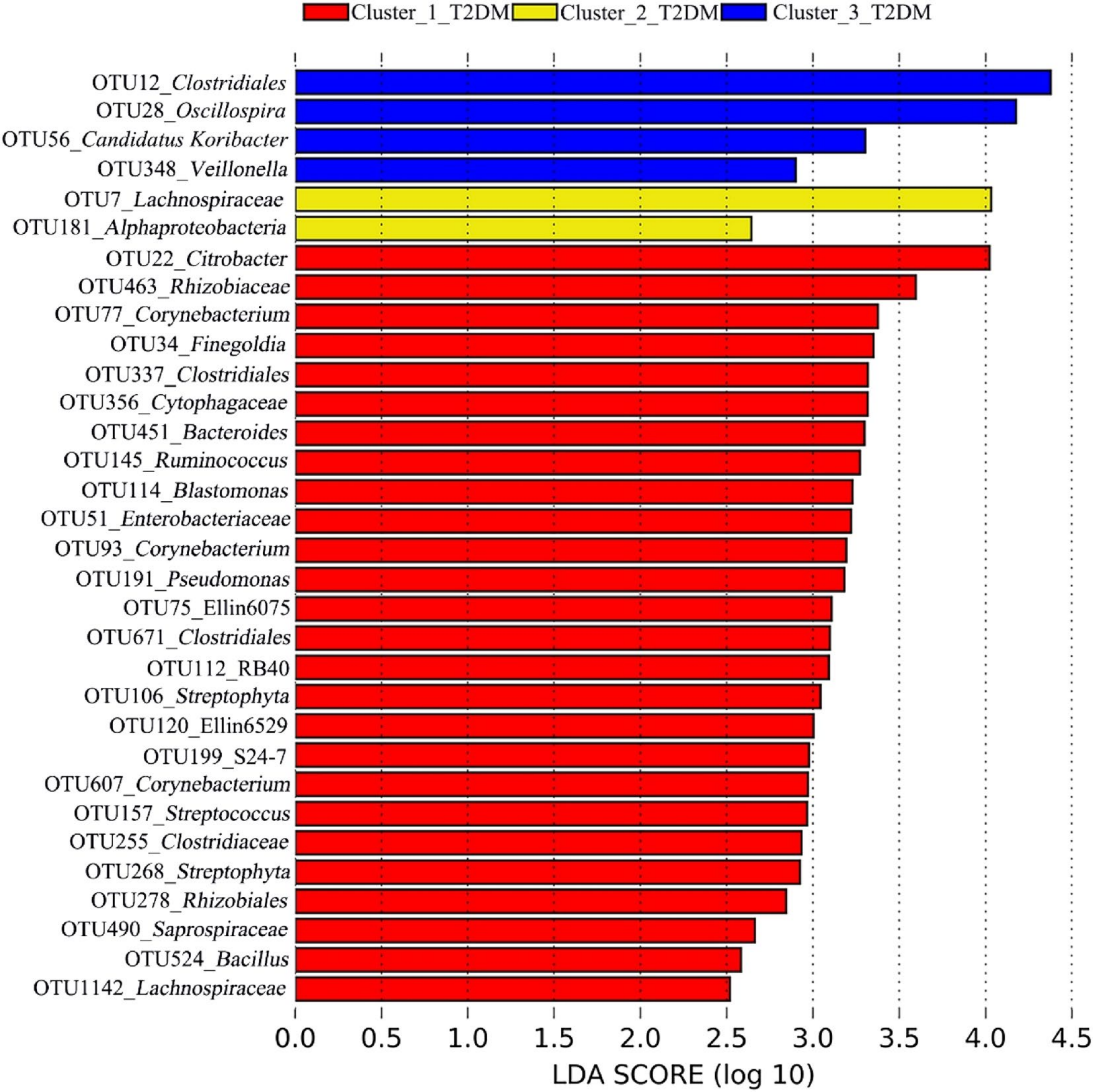
The phylotypes associated with each of the three clustered T2DM microbiomes determined by Linear Discriminant Analysis (LDA) Effect Size (LEfSe). Note: the analysis was done on the Huttenhower online software (http://huttenhower.sph.harvard.edu/galaxy).

LEfSe analysis revealed 32 representative OTUs had different associations with the three clustered T2DM microbiomes (Fig. 4). OTU12_*Clostridiales*, OTU28_*Oscillospira*, OTU348_*Veillonella* and OTU56_Candidatus *Koribacter* were the representative phylotypes more associated with Cluster_3_T2DM (Fig. 4), among which, OTU12_*Clostridiales* and OTU28_*Oscillospira* were also determined largely contributing to the dissimilarities between Cluster_3_T2DM and the two other clustered T2DM microbiomes according to the pairwise SIMPER results (Supplementary Table S3). Among the 26 OTUs more associated with Cluster_1_T2DM, OTU22_*Citrobacter*, OTU463_*Rhizobiaceae*, OTU77_*Corynebacterium* and OTU34_*Finegoldia* were the most associated representative phylotypes. In addition, OTU7_*Lachnospiraceae* and OTU181_*Alphaproteobacteria* were more associated with Cluster_2_T2DM.

No significant difference was determined in the majority of variables (e.g., urine glucose) of the T2DM patients in the three clusters (Kruskal–Wallis test, *P* > 0.5), except marriage times (Supplementary Table S4). The marriage times of Cluster_3 was significantly greater than that of Cluster_2 (Mann–Whitney test, *P* < 0.01), and similar to that of Cluster_1 (Mann–Whitney test, *P* > 0.2).

### Network and fragmentation analyses

The top 10 OTUs with most correlations in each of the three clustered T2DM microbiomes were largely distinct (Supplementary Table S5). Among them, OTU9_*Enterobacteriaceae*, OTU13_*Novosphingobium* and OTU778_*Lachnospiraceae* had most correlations in Cluster_1_T2DM, Cluster_2_T2DM and Cluster_3_T2DM, respectively.

The fragmentation analysis is used to evaluate the extent of microbiome network fragmentation. In the current study, the fragmentation level of Cluster_1_T2DM (0.611) was larger than those of Cluster_2_T2DM (0.564) and Cluster_3_T2DM (0.353). Gatekeepers were OTUs that hold together the microbiome by interacting with different parts of the network^21^, and they were determined by a fragmentation analysis in the present study. One OTU associated with Cluster_1_T2DM, i.e., OTU34_*Finegoldia*, was also determined as a gatekeeper to Cluster_1_T2DM (Fragmentation analysis, *P* = 0.023). By contrast, none of the OTUs associated with Cluster_2_T2DM or Cluster_3_T2DM could cause a collapse of networks of the two clustered T2DM microbiomes (Fragmentation analysis, all *P* > 0.05).

### Correlations between urinalysis variables and T2DM associated OTUs in each of the three clusters

**Figure 5:**
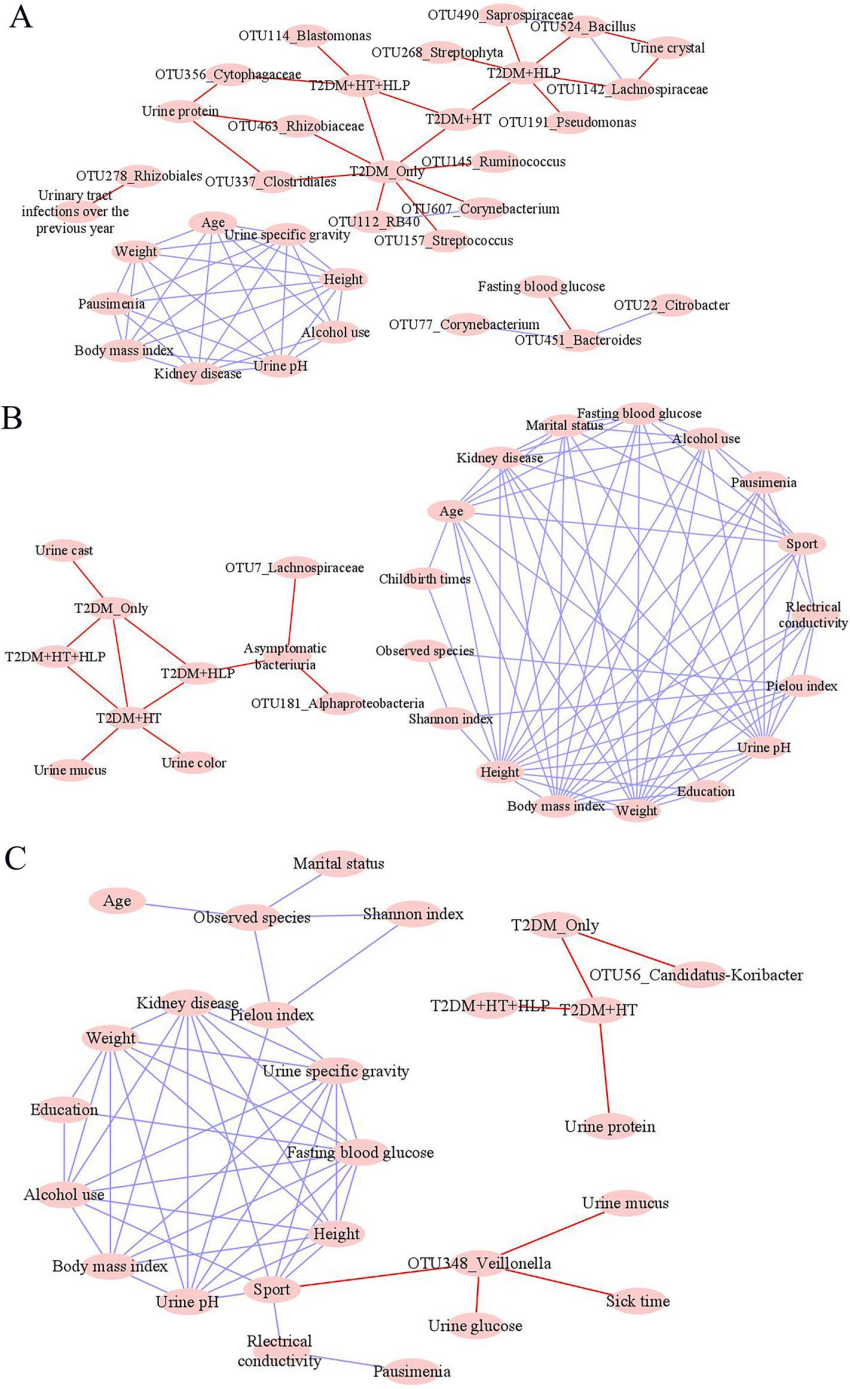
Correlations between the representative OTUs and urinalysis variables in (**A**) Cluster_1_T2DM, (**B**) Cluster_2_T2DM and (**C**) Cluster_3_T2DM. Note: the analysis was performed in Cytoscape 3.7.2. The purple and red lines represented positive and negative correlations, respectively.

Only negative correlations were determined between the urinalysis variables and representative OTUs in the three clustered T2DM microbiomes. In Cluster_1_T2DM, “Genitourinary tract infections over the previous year” was negatively correlated with OTU278_*Rhizobiales*; Urine protein was negatively correlated with OTU337_*Clostridiales*, OTU356_*Cytophagaceae* and OTU463_*Rhizobiaceae*; while urine crystal was negatively correlated with OTU524_*Bacillus* and OTU1142_*Lachnospiraceae* (Fig. 5A). In Cluster_2_T2DM, asymptomatic bacteriuria was negatively correlated with OTU7_*Lachnospiraceae* and OTU181_*Alphaproteobacteria* (Fig. 5B). Urine glucose, urine mucus and sick time were negatively correlated with OTU348_*Veillonella* in Cluster_3_T2DM (Fig. 5C).

### Functional metabolites associated with each of the three clustered T2DM microbiomes

**Figure 6:**
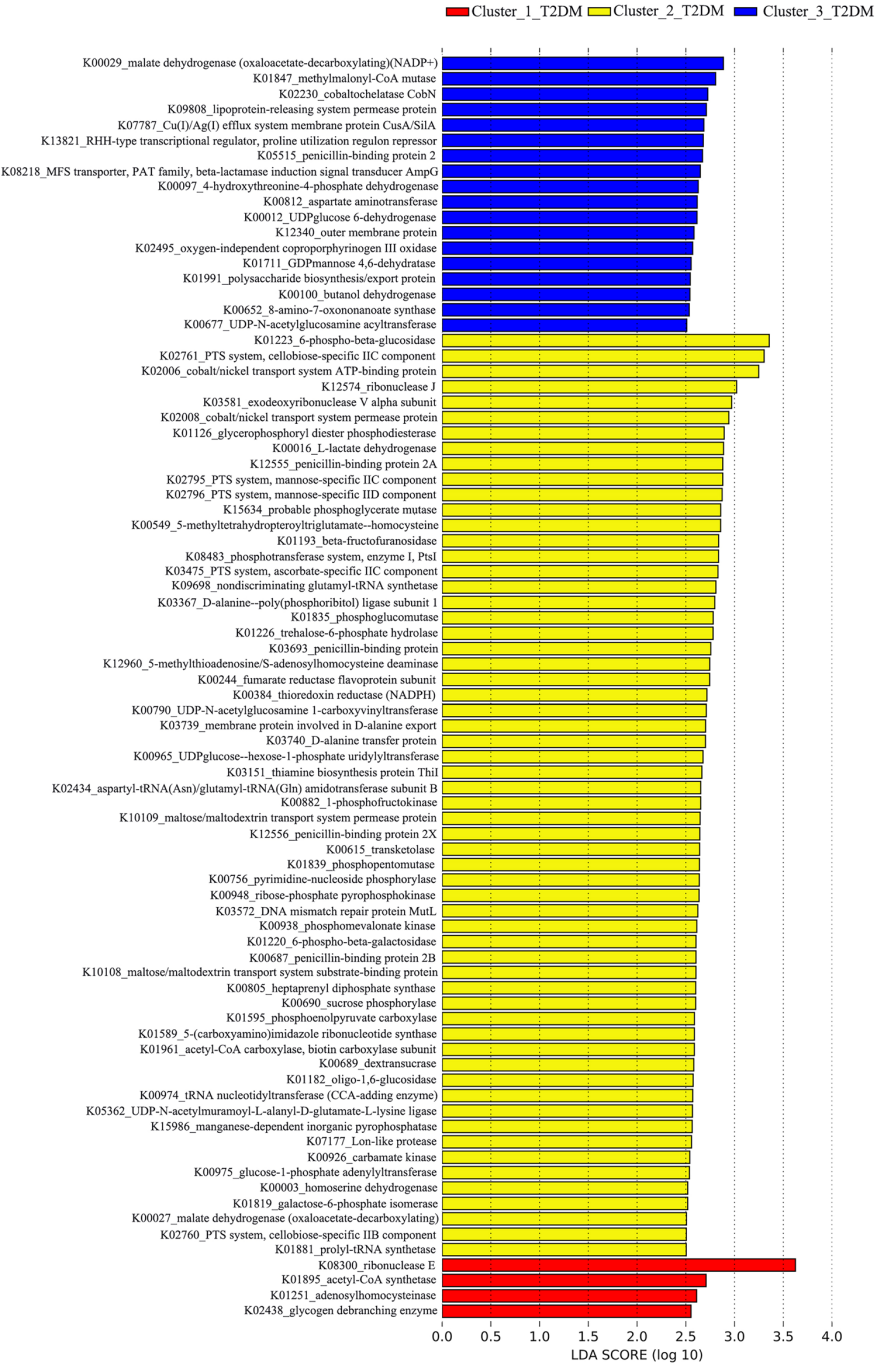
Functional metabolites associated with each of the three clustered T2DM microbiomes determined by LEfSe analysis. Note: the analysis was done on the Huttenhower online software (http://huttenhower.sph.harvard.edu/galaxy).

A total of 82 functional metabolites had different associations with the three clustered T2DM microbiomes, i.e., four for Cluster_1_T2DM, 60 for Cluster_2_T2DM and 18 for Cluster_3_T2DM (Fig. 6). Among them, K08300_ribonuclease E, K01223_6-phospho-beta-glucosidase and K00029_malate dehydrogenase (oxaloacetate-decarboxylating) (NADP^+^) were most associated with Cluster_1_T2DM, Cluster_2_T2DM and Cluster_3_T2DM, respectively.

## Discussion

Genitourinary microbiome in human beings have been well studied^36–38^. Alterations of genitourinary microbiome have been investigated in T2DM female patients and healthy female subjects, and in T2DM patients with or without other conditions (i.e., hypertension and hyperlipidemia)^9,10,12^. However, the genitourinary microbiome profiles in the T2DM patients remain poorly understood. The current study investigated the characteristics and multiple bacteria associated with different genitourinary microbiome profiles in T2DM patients, aiming to determine the phylotypes associated with the more dysbiotic T2DM microbiomes.

In the current study, the genitourinary T2DM microbiomes were clustered into three clusters, i.e., Cluster_1_T2DM, Cluster_2_T2DM and Cluster_3_T2DM, with great difference in the microbiome compositions. A group of nine OTUs associated with T2DM were significantly more abundant in one or two of the three clustered T2DM microbiomes, suggesting they had different associations with the three clustered T2DM microbiomes. *Ruminococcaceae* was enriched in the gut of mice with T2DM^39^. In the present study, OTU85_*Ruminococcaceae* was more abundant in the most dysbiotic microbiomes (i.e., Cluster_3_T2DM), suggesting *Ruminococcaceae* could be associated with the microbiomes in more than one organ of the T2DM cohorts. As for the T2DM associated OTUs with similar abundances in the clustered T2DM microbiomes, we acknowledge that whether they consistently contribute to all the three clustered T2DM microbiomes or have different functions needs further investigations.

Dysbiosis ratios of bacterial phylotypes are associated with multiple diseases^17,20,40,41^. For instance, lower cirrhosis dysbiosis ratio (i.e., abundance ratio of “good and bad taxa”) of gut microbiome was associated with more severe liver cirrhosis in the cirrhotic patients^17^. In the present study, the genitourinary MDR was significantly larger in the genitourinary microbiomes of healthy subjects than in T2DM microbiomes, suggesting the larger genitourinary MDR is associated with less dysbiotic status, which is consistent with the other disease studies^17,20^. The lowest genitourinary MDR in Cluster_3_T2DM suggests that Cluster_3_T2DM was at the most dysbiotic status, while the greatest genitourinary MDR in Cluster_1_T2DM suggested that Cluster_1_T2DM was at the least dysbiotic status. Richness and Shannon indices were both greater in the genitourinary microbiomes of healthy subjects compared with T2DM microbiomes^10^. In the current study, richness and Shannon indices were greatest in Cluster_1_T2DM, further suggesting Cluster_1_T2DM were at the least dysbiotic status among the three clustered T2DM microbiomes. The association of the three genitourinary microbiome profiles and T2DM severities/progression stages were not determined, as the information about T2DM severity and progression was not well recorded for the current study. We acknowledge that these need to be investigated in the future study.

A total of 32 representative phylotypes were associated with the three clustered T2DM microbiomes in this study. OTU12_*Clostridiales*, OTU28_*Oscillospira*, OTU348_*Veillonella* and OTU56_Candidatus *Koribacter* were associated with Cluster_3_T2DM. The three taxa, i.e., *Clostridiales*, *Oscillospira* and *Veillonella*, were also reported being associated with the other T2DM cohorts in the other studies^42–44^. *Clostridiales* was enriched in the gut of T2DM patients compared with healthy subjects^42^. *Oscillospira* was more abundant in the gut microbiomes of obese rodents compared with healthy cohort^43^. *Veillonella* was more abundant in the subgingival microbiomes of T2DM patients compared with nondiabetic subjects^44^. Among the four representative phylotypes in Cluster_3_T2DM, both OTU12_*Clostridiales* and OTU28_*Oscillospira* were also determined contributing most to the dissimilarities between Cluster_3_T2DM and the two other clustered T2DM microbiomes according to the pairwise SIMPER analyses, suggesting the two phylotypes could play vital roles in Cluster_3_T2DM. By contrast, only one of the two representative phylotypes in Cluster_2_T2DM, i.e., *Alphaproteobacteria*, was reported being enriched in the ocular surface of T2DM patients than healthy individuals^45^.

Among the 26 representative phylotypes in Cluster_1_T2DM, *Citrobacter*, *Corynebacterium* and *Finegoldia* were determined with different correlations with diabetes or relevant conditions^46–48^. *Citrobacter* was associated with asymptomatic bacteriuria in the urine of T2DM patients^46^. *Corynebacterium* was less dominant in the conjunctival microbiome of diabetes patients compared with healthy subjects^47^. Likewise, *Finegoldia* was less abundant in the gut of women with diabetes compared with healthy subjects^48^. In the current study, OTU34_*Finegoldia* acted as a gatekeeper in Cluster_1_T2DM, suggesting it may help maintain the less dysbiotic status of the T2DM patients (i.e., those patients within Cluster_1).

Fragmentation analysis has been used to investigate the fragmentation levels of microbiomes in different studies^15,21^. In the present study, the fragmentation level was greater in Cluster_1_T2DM compared with those of Cluster_2_T2DM or Cluster_3_T2DM, suggesting that less co-occurrence patterns and decreased biotic interactions in Cluster_1_T2DM.

The urinalysis variables were associated with the microbiomes or phylotypes in different hosts^49–52^. Our previous study has demonstrated that the associations between phylotypes and urinalysis variables in T2DM patients with or without hypertension or hyperlipidemia^12^. In the present study, the representative phylotypes had different associations with the different urinalysis variables within each of the three clustered T2DM microbiomes, suggesting that the representative phylotypes could be influenced by different urinalysis variables in each of the three clustered microbiomes. Likewise, the T2DM microbiomes in the three clusters were driven by relatively distinct urinalysis variables, suggesting the health status of the three clustered patients were largely different. Urine electrical conductivity and urine protein were determined to influence the more dysbiotic microbiomes (i.e., Cluster_3_T2DM) in this study, and the relevant mechanisms deserves further investigation. Confounding variables vary greatly in different studies^53–55^. While no confounding variable was determined in the current study, some alternative variables (e.g., hemoglobin A1c and medication regime) could be potential confounders to the microbiome diversity, which deserves further investigation. No significant difference was determined in the fasting blood glucose and glycosuria levels (i.e., urine glucose) of the three clustered T2DM cohorts, suggesting the two variables were not the driving factors for the microbiome compositions. A significant difference was determined in the marriage times among the three clustered T2DM cohorts, suggesting the marriage times was associated with the clustering of the T2DM microbiomes.

These results based on our 16S sequencing data could provide some useful information, but we acknowledge that whole genome sequencing and empirical metabolomics could be performed to verify the functional metabolites and identify the phylogenetics at a higher resolution in the future work.

In conclusion, there were great differences between the three clustered T2DM microbiomes, while Cluster_3_T2DM was at the most dysbiotic status. OTU12_*Clostridiales* and OTU28_*Oscillospira* were likely to drive the T2DM microbiomes to more dysbiotic status, while OTU34_*Finegoldia* could play a vital role in maintaining the stability of less dysbiotic microbiomes. The characteristics and multiple bacteria associated with the more dysbiotic genitourinary T2DM microbiomes may help with the better diagnosis and management of genitourinary dysbiosis in T2DM patients.

## Supplementary Information

Supplementary Information.

## Data Availability

The datasets for the current study are available from the corresponding author on reasonable request.
